# Understanding the treatment journey and experiences of knee osteoarthritis patients receiving standard care in Singapore: a qualitative study

**DOI:** 10.1186/s12913-026-14333-4

**Published:** 2026-03-12

**Authors:** Eugene Yong Sheng Woon, Su-Yin Yang, Rui Min Alisa Lim, Yan Qin Claudia Aw, Bryan Yijia Tan

**Affiliations:** 1 Department of Orthopedic Surgery, Woodlands Health, Singapore, Singapore; 2 Psychology Services, Woodlands Health, Singapore, Singapore; 3 National University of Singapore, Singapore, Singapore

**Keywords:** Knee osteoarthritis, Treatment journey, Treatment experience, Qualitative

## Abstract

**Background:**

A treatment journey (TJ) outlines a patient’s needs, actions, and experiences when seeking treatment. In Singapore, the quality of knee osteoarthritis (KOA) care is suboptimal. Mapping the TJ of older KOA patients can identify gaps in care influencing treatment decision, experiences, timeliness, and effectiveness for optimizing care in Singapore.

**Methods:**

This qualitative study was embedded in a larger qualitative study conducted in an urban, referral-based tertiary hospital in Singapore that evaluated and explored KOA patients’ treatment experiences of a randomized controlled trial. The larger study conveniently sampled 46 patients (22 intervention, 24 control). Semi-structured interviews were conducted in a quiet private hospital room. Framework analysis was used to analyze all 24 control patients’ transcripts. There was sufficient information power to fulfill the study’s objectives.

**Findings:**

Eighteen participants described relatively linear TJs comprising five sequential stages. Themes and sub-themes related to the stages are discussed.

**Onset:**

Participants gained awareness as symptoms impacted their daily lives, alongside sense-making processes shaped by beliefs that KOA was an inevitable consequence of ageing. A strong need to cure their condition drove informal knowledge-seeking from social networks.

**Self-treatment:**

Participants experimented with topical agents, supplements, traditional Chinese medicine, analgesics, and exercise, often delaying formal care until symptoms worsened. Symptomatic exacerbation or persistence triggered treatment-seeking.

**Primary care:**

Many participants felt dissatisfied with and dismissed from receiving limited explanations, passive management, and gatekept from specialist care.

**Tertiary care:**

Orthopedic consultations gave participants hope in conservative physiotherapy treatment and position surgery as a last resort. Positive treatment experiences and symptom improvement fostered hope, confidence, and treatment engagement. Conversely, dissatisfactory experiences led some participants to disengage from treatment and question its relevance and value.

**Post-treatment:**

Participants transitioned to self-management. Engagement in exercise and physical activity was motivated by the desire to avoid pain, deterioration, and surgery, and to maintain independence. However, inertia and perceived lack of time emerged as barriers.

**Complex treatment journeys:**

Six participants experienced complex, non-linear TJs involving multiple and/or repeated healthcare touchpoints that caused confusion and frustration.

**Discussion:**

Improving community knowledge of KOA could meet older KOA patients’ strong need for treatment information, which influences sense-making, self-efficacy, decisions, and eventually treatment length, complexity, effectiveness, and experience. Healthcare providers could provide informative communication and feedback that fulfill their patients’ expectations for both information and care, as well as their need for hope confidence, reassurance, help, and relief. Inertia and a lack of time can be counteracted by providing meaningful, manageable, and adaptable care.

**Conclusion:**

Inadequate communication from healthcare providers are pain points in a TJ. Pain history and severity, and degree of physical functioning and KOA knowledge can influence a TJ’s length and complexity.

**Trial registration:**

This study, embedded in a larger study that qualitatively evaluated data from a randomized controlled trial (NHG DSRB ref no: 2020/00067) Tan et al. (BMC Musculoskelet Disord. 21(1):684, 2020) that explored the treatment experiences of KOA patients, is not a clinical trial (clinical trial number: not applicable).

**Supplementary Information:**

The online version contains supplementary material available at 10.1186/s12913-026-14333-4.

## Introduction

 Knee osteoarthritis (KOA), the most common form of arthritis, is characterized by knee pain, joint stiffness, inflammation, and lower limb weakness [1]. Global prevalence of individuals aged ≥ 40 with KOA is estimated at 22.9% of the world’s population [2]. Prevalence rates in Asia (19.2%) are higher than rates reported for North America (15.8%) and Europe (13.4%). Risk of developing KOA increases with age; with KOA being the leading cause of pain and disability among older people [3]. With a fast-aging population and increasing rate of obesity worldwide [4], prevalence rates of KOA are expected to increase reciprocally.

The patient journey outlines an individual’s diagnoses, actions, treatments, and interactions with healthcare providers throughout the various stages of a disease [5]. It commences at the onset of symptoms, prior to one’s self-initiated or otherwise self-management or interaction with healthcare touchpoints. Treatments for KOA have traditionally relied on pharmacotherapy as the first line of treatment to afford pain relief, often negating the added value of exercise recommendations for functional improvement. Hence, whilst Osteoarthritis Research Society International guidelines recommend structured land-based exercise programs and education as an essential part of KOA treatments, recommendations based on these guidelines in clinical practice have been mixed. Despite guidelines, more than 60% of patients globally receive suboptimal care [6]. In Singapore’s context, it is notable that in spite of the island nation’s an extensive primary care network of about 2,000 private general practitioners, 26 polyclinics (‘one-stop’ healthcare centers that provide primary medical treatment, preventive healthcare, and health education), and a tertiary care network work of eight general hospitals, a recent study found the quality of KOA care in Singapore to be suboptimal [7].

Literature suggests some individuals did not seek timely treatment for their condition due to (1) the belief that knee pain is a natural part of ageing [8], (2) prioritizing other co-morbidities over knee pain [9], (3) seeking help only when symptoms greatly affect daily activities [10], or (4) relying on traditional treatment for temporary symptom relief [9]. Furthermore, long wait times for [11] and time-limited consultations are major healthcare services issues associated with poor treatment satisfaction and compliance [12], and subsequently increased healthcare utilization [13]. These demonstrated patient concerns can be identified at every touchpoint; improve the relevance of policies and quality of care in institutions and the community [14] to meet patient needs; and improve patient experience—a key quality indicator for healthcare and positively associated with clinical effectiveness [15]. Understanding potential patient barriers that contribute to their presentation and decision-making process through the treatment journey is necessary to support patients navigating the health system for timely and effective care.

In recent years, patient journey mapping is a fast-growing method for learning how patients enter, experience, and leave health services [16]; understand patients’ needs; provider-patient interactions; care/intervention transitions, integration, simplification, and design [17]. In addition to non-surgical treatments provided at the polyclinics and general hospitals, Traditional Chinese Medicine (TCM) are readily accessible to Singaporeans, with 20% using it annually and 40% used a combination of Western medicine and TCM for the same condition [18]. As such, driven by needs, symptoms, diagnoses, and treatment experiences and satisfaction, as well as access to a various treatment modalities, individuals could have complex, non-linear journeys by entering and exiting touchpoints at any stage, while requiring differing levels of care at other points [19].

This study aims to (1) develop a deeper understanding of the treatment journey and experiences of older KOA patients receiving standard care to (2) inform improvements for more optimal KOA care in Singapore.

## Methods

### Study design and setting

This descriptive qualitative study, which seeks to understand the treatment experiences, journey, and needs of older KOA patients seeking standard treatment was embedded in a larger a priori qualitative process evaluation study of a community-based intervention randomized controlled trial (RCT) [20] against usual care for KOA patients. The larger study was conducted in an urban, referral-based tertiary hospital in Singapore.

### Sampling strategy and participants

The larger qualitative study conveniently sampled 86 eligible patients: 55 patients responded, nine (16%) declined due to a lack of interest, and 46 (84%) provided data. Among the 46 individuals who provided data, there were 22 intervention (47.8%) and 24 (52.2%) control patients. As the larger qualitative study was conducted between April and July 2020, its sampling strategy was constrained by the older patient population’s safety concern with COVID-19 and the 2-month “circuit breaker” period in Singapore.

As this study seeks to understand the treatment journey of patients receiving standard care, data from the 24 conveniently sampled control patients was used. The sample consisted of 16 females and eight males, with a mean age of 68 (Table 1). All patients were (1) at least 45 years; (2) had a clinical diagnosis of KOA (NICE guidelines) [21] in at least one knee (i.e. patients who had undergone total knee arthroplasty in one knee and has clinically diagnosed KOA in another knee are eligible for inclusion.); (3) were ambulatory in the community with or without a walking aid; (4) prescribed conservative outpatient treatment in the hospital; (5) and were conversant in either English or Mandarin [22].

**Table 1:** Description of interviewees’ baseline characteristics

Subject (*n* = 24)	Sex	Race	Age	Language Spoken	Years of Education	Employment Status	KL Grade	KOOS-4
P01	M	Chinese	65	English	10	Retired	3	61.93
P02	F	Chinese	71	English	0	Full-time	4	51.79
P03	M	Chinese	53	English	10	Retired	3	52.42
P04	M	Chinese	65	English	10	Retired	3	64.89
P05	F	Chinese	75	English	13	Full-time	3	67.09
P06	F	Indian	62	English	6	Full-time	3	58.65
P07	M	Chinese	77	English	10	Retired	3	59.36
P08	F	Chinese	71	English	Missing data	Retired	4	71.82
P09	F	Chinese	62	English	12	Retired	3	45.45
P10	F	Chinese	73	Mandarin	13	Retired	3	68.86
P11	M	Chinese	62	English	13	Retired	3	63.67
P12	F	Chinese	82	Mandarin	6	Retired	3	75.32
P13	F	Chinese	72	English	10	Retired	2	67.79
P14	F	Chinese	68	English	13	Retired	2	71.2
P15	M	Chinese	82	Mandarin	0	Full-time	3	61.28
P16	F	Malay	70	English	10	Retired	4	35.39
P17	M	Chinese	74	Mandarin	6	Full-time	3	70.92
P18	F	Chinese	64	Mandarin	10	Retired	2	56.1
P19	F	Chinese	75	English	16	Homemaker	3	57.37
P20	F	Chinese	66	Mandarin	10	Full-time	2	49.42
P21	M	Chinese	62	English	13	Retired	4	58.28
P22	F	Chinese	60	English	10	Retired	2	50.17
P23	F	Chinese	72	English	10	Full-time	3	52.79
P24	F	Chinese	51	English	10	Retired	3	30.19

*Kellgren Lawrence (KL) grade

Males = 8 (33.3%), Females = 16 (66.7%)

Chinese = 22 (91.6%), Malay = 1 (4.2%), Indian = 1 (4.2%)

Age (mean) = 68

English = 16 (66.7%), Mandarin = 8 (33.3%)

Years of Education (Mean) = 9.2

Full-time = 7 (29.1%), Retired = 16 (66.7%), Homemaker: 1 (4.2%)

KL 2 = 5 (20.8%), KL 3 = 15 (62.5%), KL 4 = 4 (16.7%)

KOOS-4 (KOOS Pain, Symptoms, Sports/Rec, and Quality of Life) (mean) = 58.42

### Data collection and management

The interview questions for this study were embedded in the interview guide of the larger qualitative study, which was pilot tested before implementation. Interviews were arranged after participants gave informed consent. Two participants were accompanied by a family member, who neither influenced the participants nor the interview. There was no prior relationship between the interviewer and participants [22]. The participants were informed that the purpose of the research was to understand their treatment journey, experiences, and needs in order to improve health services. During the data collection period, no participant was scheduled for knee arthroplasty surgery.

This study’s data collection and management processes are similar to another embedded related study [22]. Semi-structured, face-to-face, audio-recorded interviews were conducted in either English or Mandarin, between April 2020 and July 2020, in a quiet private room in the hospital, by EWYS. The interviews lasted between 30 and 80 min. Field notes were made during or after the interviews. The interviews were transcribed verbatim, translated from Mandarin to English where necessary, and de-identified with the participant’s assigned subject code. The transcripts and translations were compared against their corresponding recordings for accuracy. The transcripts were not returned to the participants for comments and correction as they were contacted only once for the study.

### Research team and contextual reflexivity

EWYS is a male bilingual researcher with a Master’s degree, five years of qualitative research experience, with an interest in degenerative joint diseases and healthcare research. SYY is a highly experienced female senior researcher in qualitative methodologies with a PhD in clinical psychology, while BYT is an experienced male researcher with a PhD and an orthopedic surgeon. AL and CA were female undergraduate interns who were trained and closely supervised by EWYS in coding qualitative data and creating individual patient treatment journeys. SYY and BYT, being medical practitioners in an urban, referral-based tertiary hospital in Singapore, possessed deeper contextual knowledge and understanding of the nation’s healthcare system’s systemic limitations and issues, which provided a much-needed clinical lens during data interpretation. Although SYY, BYT, and EWYS are experienced researchers CA and AL were encouraged to openly voice their thoughts and opinions during discussions. All members of the research team are locals with contextual knowledge and experiences of Singapore’s healthcare system’s mechanisms, which facilitated the comprehension, analysis, and interpretation of the results. The study’s data were used to objectively moderate biases (e.g. empathizing with systemic limitations) during data interpretation discussions.

### Methodological reflexity

Due to the semi-structured nature of the interviews, older participants may not narrate their treatment journeys in chronological order. This may be due to the participants’ interest, recall bias, and perceived relevance/significance of particular experiences or the interviewer’s follow-up questions. Hence, EWYS decided is a need to chronologically plot each participant’s treatment journey according to their narrations instead of following the sequential stages suggested by literature. This prevents analytical bias, as well as allowing common patterns and unique divergences to emerge when cross-comparing individual journeys. Moreover, review of literature provided awareness of the treatment journey stages, as well as conventional KOA treatment options. As such, care was taken to avoid allowing theoretical knowledge from influencing the interview questions. Assumptions arising from the participants’ narrations during the interviews were clarified by asking “Based on what you had shared earlier, do you mean…?” and “Am I right to understand that you are trying to mean…?”.

### Data analysis

This study, similar to the larger qualitative process evaluation study that it was embedded in, employed framework analysis. Its spreadsheet approach allowed the study to use Microsoft Excel to manage, structure, and analyze the dataset [23]. The study’s use of framework analysis was replicated from another embedded related study [22].

**Fig. 1:**
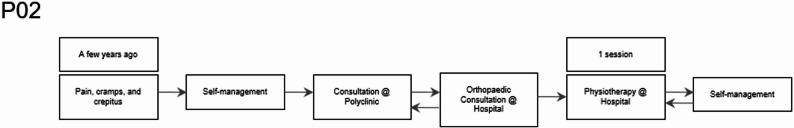
Example of a mapped patient treatment journey

Five transcripts were randomly selected and three researchers (EYSW, AL, and CA) independently coded them line by line. The transcripts were coded deductively and inductively. Deductive coding used pre-established codes and categories gleaned from literature, which informed the treatment journey stages a priori. Guided by the principles of grounded theory, inductive coding adopted an open, comparative, and iterative approach to elicit substantive codes [24] to describe the themes. The coders compared their codes to achieve shared and rigorous understanding and agreement on the codes and their reflection of the data before the codes were applied to the remaining transcripts. Coding and recoding were cyclically repeated to refine the existing and emergent codes and sub-themes as their meanings become clearer and precise. EYSW was the primary coder, while AL and CA were the secondary coders, for all the transcripts. AL and CA chronologically mapped the treatment journey of each participant (refer to Fig. 1) by following their narratives closely, EYSW then verified the accuracy of each mapped treatment journey. All authors examined disparities in the interpreted data with open minds and from different perspectives to retain richness of insights before concurring on the emergent themes and constituent sub-themes and codes that mirrored the data. Supplementary Table 3a (refer to Additional file 1) provides an overview of the subthemes, which were deductively coded and categorized according to pre-established concepts gleaned from established literature. The reporting of this study is in accordance with the COnsolidated criteria for REporting Qualitative research.

This study uses Guba and Lincoln’s [25] criteria for establishing trustworthiness (Table 2).

**Table 2:** Strategies for establishing trustworthiness

Criteria	Strategies
Credibility	- Had prolonged engagement (lasting between 30 and 80 min) with participants to build rapport and thoroughly understand the context - Semi-structured interviews are used to allow participants to freely express and fully elaborate on their experiences and perspectives - Interviewer clarified all assumptions and the participants’ expressed and intended meanings throughout the interview by using prompts such as “What do you mean by that?” to establish accuracy of understanding of elaboration
Dependability	- Provision of thick descriptions of the data, study context, and participant characteristics
Transferability	- Interviewer took care to strategically follow-up on all possible questions to obtain complete accounts of experiences and perspectives (i.e. each interview is saturated) by using prompts such as “Please tell me more about it” to establish thoroughness of elaboration
Confirmability	- Data is analyzed with an open mind - The primary coder’s perspectives, beliefs, preconceptions, experiences, and analytical blind spots are mitigated via analyst triangulation with co-authors who understand and interpret the data in multiple/different ways. During the analyst triangulation meetings to resolve disagreements, the team opt to reflexively interpret the codes, categories, and emergent themes to retain richness of insights.

### Data and analytical sufficiency

Although saturation serves as a guideline for methodological rigor in qualitative research, there is uncertainty in operationalizing, determining, and declaring saturation [26, 27] Some suggested that the inherent complexity and subtlety of human experiences makes saturation extremely difficult [28]. Acknowledging the limitations of its sampling strategy, this study prioritized data sufficiency via information power (i.e. strong relevance of a relatively homogeneous sample to the research [28], as well as breadth and richness of insights). Based on the strategies employed for establishing trustworthiness (Table 2), as well as multiple analyst triangulation discussions, the study team determined that there was adequate information power [29] from the 24 transcripts for reasonably clear and good comprehension of the codes and development of themes required to answer the study’s research questions (hybrid data and thematic sufficiency [30]).

### Ethics approval

This study is embedded in a larger qualitative study [20] and shares that study’s ethics approval (National Healthcare Group Domain Specific Review Board (NHG DSRB) ref no: 2020/00067). This study adhered to the Declaration of Helsinki.

## Results

The authors recruited 24 participants: 16 females (66.7%) and eight males (33.3%); 16 English-speaking (66.7%) and eight Mandarin-speaking (33.3%); 22 Chinese (91.66%), one Malay (4.17%), and one Indian (4.17%); with a mean age of 68. Among the participants, 16 are retired (66.7%); seven in full-time employment (29.2%), and one homemaker (4.1%). All participants experienced symptoms of KOA for at least six months, with some indicating longer durations of between three years and a decade. Table 2 summarizes the participants’ characteristics.

**Fig. 2:**
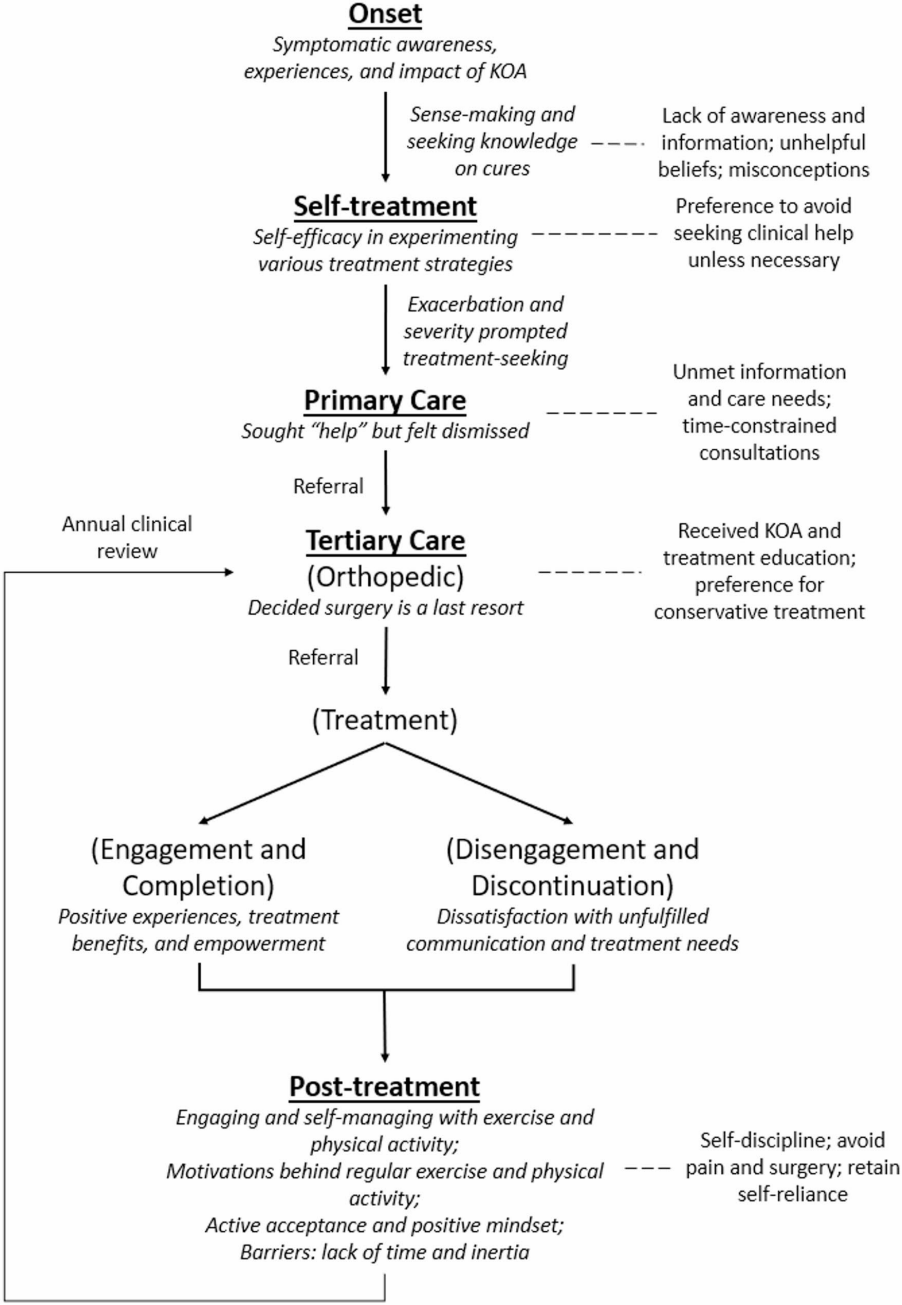
Graphical representation of a typically linear patient treatment journey

Comparison of 24 chronologically-mapped individual treatment journeys showed 18 participants had relatively linear treatment journeys consisting of five sequential stages: Onset, Self-treatment, Primary care, Tertiary care, and Post-treatment. Figure 2, a graphical representation of the linear treatment journey that was typical for most participants, was constructed utilizing the five stages (in bold) and accompanied by their corresponding themes (italicized). Six participants had complex treatment journeys. Regardless of their treatment journey, all participants eventually enter Tertiary care and finally the Post-treatment stage where they continue to self-manage their condition and symptoms.

### [Stage 1] onset

#### Symptomatic awareness, experiences, and impact of KOA

Participants began to pay more attention to their condition when they persistently experienced pain, joint stiffness, and difficulty in walking or rising from a squatting or sitting position. A few were caught offguard by an unexpected onset of debilitating severe pain when they were “*trying to climb the steps in my house and then suddenly it just hurts… and you just couldn’t move!*” (P14) or “*I was in the bus… I get up and the pain was very sharp. I tried to walk*,* but I can’t… that’s the worst pain I had in my life!*” (P06).

Pain affected the participants’ activities of daily living. Majority would attempt to complete their daily tasks independently despite pain, while a few required their children’s assistance with strenous actitivies like carrying heavy groceries and household chores (e.g. hanging wet clothes out to dry). Their ability to walk for longer duration or distances were also affected by the pain and challenges caused by built environment factors like stairs and slopes.

#### Sense-making and seeking knowledge on cures

Many participants primarily believed their condition was caused by wearing out of the joint because “*age is catching up*,* those bones wear and tear*” (P13) whereby “*degeneration is still degeneration*,* nothing can be done*” (P20). Some male participants also attributed their condition to injuries from sports and activities during military service.

Participants asked family, relatives, neighbors, friends, and colleagues for ways to cure their pain. One stated, “I will listen and pay attention to find out which doctor is good, or how to treat the knee. It’s good to understand these things… so you will know what you need to do” (P10). A few turned to the internet and social media for ideas. Many learned about the experiences of others who had undergone knee surgery. Of interest are those who heard positive outcomes yet were not swayed to undergo surgery. A few reported being at a loss as they “don’t know what to do… because I don’t know what happened” (P06), “cannot find any solution” (P21) because it “never happened before, so I don’t know why suddenly this happened” (P12).

### [Stage 2] self-treatment

#### Self-efficacy in experimenting various treatment strategies

Participants, believing their symptoms would eventually improve and disappear after self-medicating, would try and evaluate the effectiveness of a variety of strategies such as topical applications of oils and creams, supplements, painkillers, TCM, and exercises and physical activity to manage their pain. Some participants continued to use some of these strategies in the later stages of their treatment journey whenever they experience symptoms. Most participants preferred to self-manage and delay seeking clinical diagnosis and care for as long as possible until persistent or worsening pain triggered the need to do so.

Topical application of medicated ointments and analgesic creams forms the default self-treatment strategy adopted by participants “*use some ointment to rub on my knee*,* hoping that I will be able to stand up and walk*” (P22). However, it was “*not that effective because it didn’t drive the pain away.”* (P11). Participants prefer to tolerate pain and “*avoid taking painkillers like Panadol because I know that it’s not good for the body*” (P01), fearing painkillers will harm their liver and kidneys, or cause addiction. Although, some will medicate when pain becomes unbearable, they still “*try to avoid painkillers. I don’t take very often unless I can’t stand the pain… I try to control the pain*” (P06).

Regarding glucosamine consumption, participants recounted being advised by their tertiary care orthopedic surgeons to cease consumption as it is unlikely to benefit their condition. Yet, a few continued consuming glucosamine as they saw no harm in it despite its lack of effectiveness.

Half of the participants tried acupuncture because they initially believed “*after acupuncture*,* your leg will feel very good*” (P17). However, they were disappointed by its temporary effectiveness “*last*(s) *about two to three days… I don’t want a temporary cure. I just want something that really can solve my problem*” (P22) and felt it “*is a waste of money. Because the acupuncture is not only do once then ok … must do it often*,* if not it will be as usual: cannot move*” (P20). Another half tried exercise as they “*know physical activities can help*” (P22) with their pain, one shared, “*I start brisk walking*,* I do swimming… it’s not so painful anymore!*” (P22).

#### Exacerbation and severity prompted treatment-seeking

Participants expressed they would continue to cope independently for as long as possible until their coping strategies fail to alleviate their symptoms. They delayed seeking treatment because they *“didn’t realize the severity. I thought it was a minor injury so I tried to self-medicate first”* (P01) or expected *“the pain would go away after a few days”* (P11). Symptomatic persistence and exacerbation then increased their perceived pain severity, which prompted them to seek medical help.

At the beginning, I felt it’s (pain) still bearable because this is on and off… this time, the pain continues and didn’t get better. So, I felt it’s quite serious and need to see the doctor. – P18

About 6 months, I didn’t see any doctor. Once I see like more painful is when I went to see doctor. – P16

### [Stage 3] primary care

#### Sought “help” but felt dismissed

Several participants felt resigned to their diagnosis and disappointed with the inadequate information and treatment. They gave similar accounts like “the doctor just said it was aging, gave me glucosamine and painkillers. Nothing else” (P18) and “the doctor didn’t say anything in general, just only ‘it’s degeneration’” (P19). Some expressed their disatisfaction with the care they received, “I was quite disappointed in one way. I cannot get proper advice… I don’t get anything, any advice, what they give is only medicine and then ask you to come back again!” (P24). They explained that the doctors “are very busy, so they don’t feel like talking much to you. You ask one question they answer you one question… I like to know (more) but I know that they can’t tell much; outside so many patients waiting for them.” (P19). A few expressed their disatisfaction with the need to make repeat visits in order to obtain a referral to the hospital.

The first time, I requested the doctor to give me a referral letter to see the specialist but he refused… I was quite disappointed because it was so painful but the doctor just said it’s arthritis, degeneration and that there’s no treatment. I went home and complained to my family and they said polyclinic is like that. – P22

(the doctor will) just state your condition, give you medicine and that’s it. If you still don’t feel well, come back one week later. It’s only when you come back for a second visit that they will start the referral process- P01

None of the participants indicated receiving a referral from their primary care doctors to physiotherapy treatment at primary care.

### [Stage 4] tertiary care

#### Orthopedic consultation: decided surgery is a last resort

Upon receiving education on KOA and its treatment options, many participants opted for conservative treatment and either believed or hoped that their condition is “*curable. I don’t need to go for kneecap replacement*,* so by doing* (physio)*therapy*,* it can help me to heal*” (P03). The participants expressed apprehension towards surgery due to their age, as well as the procedure’s cost, risk of failure, and arduous post-surgical recovery. Even though some previously considered or urged by others to undergo surgery, participants consider surgery as the last resort whereby “ *I have not reached the point whereby it’s so painful until I cannot walk. I’m trying to defer it… I’m not ready to do it*” (P09). The participants’ reasons for avoiding or considering surgery are presented in Supplementary Table 3b (refer to Additional file 1). All participants received a hospital referral to physiotherapy treatment; one requested for injection treatment (P15), another refused a referral to a dietician (P08).

#### Treatment engagement and completion: positive experiences, treatment benefits, empowerment

Positive experiences (e.g. empathy, concern, and reassurance) with their physiotherapists, as well as experiencing improvements in pain and physical functioning led participants to develop confidence and hope in treatment and recovery, and encouraged treatment engagement, which is exemplified by the following quotes:

The physiotherapist gave the confidence and when he understood that I could not do certain things, he tried to find alternative ways for me to do… I am happy with how he’s trying to help. So, it’s good… I will go back and try the exercises because I know that I do will really help me and I really need those exercises. I am quite confident that this round of physiotherapy is going to give me better results… now my knee is not that painful anymore. I’m walking faster and better. – P09

The physiotherapist was professional, friendly, and seemed to know what she’s doing. She took measurements to check my knee to see how it was, then recommended exercises to help alleviate the pain… after talking to the therapist, I understand that the amount of recovery will most probably be equivalent to the amount of effort I put into the exercises. I followed the prescribed exercises strictly… The exercises helped quite a bit. Some of the mobility has returned to my knee, and the pain has lessened a lot. There are also certain things that I can do, such as cycling and stair climbing. – P11

Participants also emphasized on the need for treatment engagement, one shared, “the physiotherap(ist) gives you the skill… a technique. Problem is whether or not you want to do it, cannot blame others… It is evident that I must rely on myself… After the first session, I exercised already, then start to get better… there is hope” (P20).

#### Treatment disengagement and discontinuation: dissatisfaction with unfulfilled communication and treatment needs

Some participants felt the exercises prescribed were mild, repetitive, unhelpful, or unsuitable due to pain. One described the treatment as “*doing a warmup before we exercise… I do not have that true feeling physiotherapy will help… definitely no. I feel that it is not helpful.*” (P01). They were strongly dissatisfied with the limited communication from their therapists, whereby “*after I’m done with the exercise* (sessions), *that’s it*,* I go home. No connectivity*,* no further feedback*,* advice or concern*” (P22). A participant explained how a lack of education from therapists cause feelings of anxiety due to one’s unfulfilled need to better understand his current condition and future progress, “*Each time I come to see you*,* this guy looks at your case and say ‘the physiotherapist follows a set of exercise repetitively: this is like that*,* now we do the same set’*,* without explaining the benefits… you don’t know how effective it is… you put me through physiotherapy and not telling me how much improvement I am going to (have)… When we are not feeling well*,* we want to improve as fast as possible*” (P01). The above disatisfactions led these patients to question the need to continue with their physiotherapy sessions, “*… these physio sessions*,* it’s very time wasting*,* not productive…effectiveness is not there… so*,* what is the benefit?*” (P01).

### [Stage 5] post-treatment

Although all 24 participants subsequently entered the post-treatment phase, a few indicated having annual reviews with their orthopedic surgeons. Participants who experienced improvement after treatment could often go about their daily activities.

#### Adhering and self-managing with exercise and physical activity

While some participants continued with their physiotherapy exercises, several engaged in exercises and activities that suited their interests, preferences, needs and lifestyles. This enabled them to engage in regular physical activity albeit with caution, “*I know certain parts of my body are already already giving way*,* so I cannot overdo it such that I have injuries elsewhere. I just follow the regime given to me strictly*” (P17). The sub-themes and corresponding quotes are presented in Supplementary Table 3c (refer to Additional file 1).

#### Motivations behind regular exercise and physical activity

The desire to avoid pain, deterioration, and surgery motivated most participants to persist with regular exercise and physical activity. They shared, “*if I stop exercising*,* I’m afraid it* (pain) *will return. To prevent the pain from returning*,* I tell myself I need to persevere in my exercises*” (P18) and “*I’m confident if I keep on doing*,* the pain will be gone… don’t just give up! I always think the exercises I do today must be better then what I did before… Improve faster depends on myself”*. They believe exercise can improve their knee, overall health, and energy levels, “*I think Zumba and chair exercise help me to improve my leg and health*” (P05). Participants also believe being self-discipined in regular exercise and physical activity is essential for maintaining independence within the community, *“this needs a lot of self-discipline with the exercises… You just have to tell yourself to pick it up and work on it”* (P14). One elaborated, “*I must stay strong*,* healthy because I don’t want to be a burden to my husband*,* children… I’m disciplined enough to do* (exercise) *on my own*” (P22).

#### Active acceptance and positive mindset

Some participants expressed acceptance of KOA and pain, and are aware “the pain is still there… it’s also normal because everything comes back to exercise, medicine, and ageing… It’s to do with your thinking, your life still have to carry on. There’s no cure for this sickness… doesn’t mean everything can be cured, sometimes you just have to accept it” (P07).

#### Barriers: lack of time and inertia

Competing priorities and time needed for exercise or physical activity amount to a lack of time (actual or perceived), which is a barrier to self-maintenance of regular exercise or physical activity.

Inertia is another barrier whereby participants felt “*very lazy… you don’t feel like doing anything*,* then doing less and less*” (P19). One participant provided in-depth insight into how engagement in exercise and physical activity is affected by both inertia and a lack of time:

We’re supposed to do 3 times per day, I say ‘no, I can’t because of time constraints, who has time to do 3 times a day?’… when you are lazy, you tend to just neglect… when you do exercises it’s not immediate, it’s a long haul thing,… I feel that this needs a lot of self-discipline with the recommended exercises… When there’s no supervision, you think you have got more important things to do than this. (P14)

### Complex Treatment Journeys

Conversely, among the 24 participants, six had longer and more complicated journeys involving multiple or repeated visits to the emergency room, primary care, as well as seeking diagnoses and treatments from different public tertiary and pivate hospitals (refer to Supplementary Fig. 1, Additional File 1).

Participants’ recounted being confused by their multiple different dignoses; dissatisfied with a lack of advice and effective treatments; feeling helpless against the healthcare system; and self-managing with passive strategies like glucosamine and TCM while being on passive annual follow-ups for several years. Almost all of these participants stopped their search for a diagnosis and cure when they received a definite diagnosis and experienced symptomatic improvement after attempting treatment at the hospital.

I went to see three or four private doctors, and every one of them said different things. So before coming here, I also go to some other place to check my blood vessels. In the end, I still do not know what condition I have!… they’re quite careless, different hospitals say different things, like something is blocked. In the end, I came to [this hospital] and did physiotherapy, so I stopped going to the private doctors… the private doctor also referred me to some other private doctors, so I can’t remember which private doctor who referred me here… (P10)

I was quite disappointed in one way, how should I put it, I cannot get proper advice from seeing the doctor (from a private hospital). So there is one stage I have to depend on a walking stick, and I really cannot walk, and I was so upset! I even went to those specialist, I pay more for that to go through the treatment but it seems to be the same… Actually just MRI (at the private hospital), then some as usual ah you know when I go for the Xray, the knee all this already joint, the knee bone or something like that… (P24)

Although all six patients had relatively positive experiences with their physiotherapist and expressed they were self-managing well with personal exercise and physical activities, only five experienced improvements in pain, physical functions, and mobility.

## Discussion

Interviews with 24 older KOA patients shed valuable insight into their cognitive processes (beliefs, mindsets, and decisions), behaviors (sense-making, information-seeking, and self-treatment), and experiences (symptoms, healthcare professionals, treatment) navigating treatment for KOA. A typical older KOA patient’s treatment journey, mirroring the sequence of patient interactions with healthcare services [19], appears relatively linear with five stages: Onset, Self-treatment, Primary Care, Tertiary Care (Orthopedic and Treatment), and Post-treatment. Nevertheless, patients with complex treatment journeys involve consultations with different specialists from public and private hospitals due to dissatisfaction with their diagnosis, advice, treatment, and/or outcome as main reasons. The complex treatment journeys depicted in this study showed patients can enter and exit touchpoints at any stage, while requiring differing levels of care at other points [19].

As KOA is currently incurable, the treatment journey would become dormant if the patient is self-managing well and does have a need for any healthcare services. Otherwise, it would remain active if a patient is following up with an orthopedic surgeon or self-managing with passive modalities like acupuncture or massage therapy. A dormant treatment journey could resume if symptoms worsened and require clinical investigation and/or care.

Results from this study suggest patient dissatisfaction with the Primary Care and Tertiary Care (Treatment) touch points. The results indicated patients are likely to drop out (defined as patient withdrawal from an exercise program before its completion) [31]—of physical therapy at the Tertiary Care (Treatment) stage due to therapist and treatment factors. This is consistent with Spitaels’ et al.’s study [32], which found healthcare professional factors were the most prominent barriers to treatment completion [33, 34]. In this study, discontentment with therapists and exercise treatment are the main motivators of self-discharge. Symptoms improvement and having the confidence to self-manage are rarely the reasons to stop treatment [35–37]. It would seem that efforts should be concentrated at Primary Care and Tertiary Care (Treatment) to improve patient engagement, experience, and outcomes.

### Mediating effects of self-efficacy

Throughout the Onset, Self-treatment, Tertiary Care (Treatment), and Post-treatment stages, several patients demonstrated intrinsic self-efficacy via their behavior (i.e. information-seeking, varied self-treatment strategies) and attitudes (i.e. recognizing the need for and importance of treatment engagement – defined as a “co-constructed process and state…of gradually connecting with each other and/or a therapeutic program, which enables the individual to become an active, committed and invested collaborator in healthcare” [38] –) for regular physical activity to maintain independence. It is possible that older knee OA patients’ self-efficacy appears to be driven by their preference to be independent and low perceived severity of their condition (i.e. delaying physician consults until symptoms significantly affected their daily lives [39], as well as their belief that knee pain is a natural result of ageing), which led them to self-source ways to manage their health problems [40] by seeking advice from their social network and utilizing various forms of accessible and affordable care [41] that could extend or complicate their treatment journey.

This study showed achieving mastery and receiving positive reinforcements by healthcare providers [42] improved self-efficacy during treatment, while positive physiological improvements in mobility, pain, and function, as well as the desire to avoid pain, deterioration, and surgery fueled self-efficacy in self-managing post-treatment. Yet, self-efficacy can be threatened by internal and external barriers such as inertia and lack of time. As such, therapists could consider exploring ways to help older knee OA patients (according to their needs, preferences, goals, and personality traits) to routinize or make a habit out of exercise and physical activities to enhance self-control (i.e. an individual’s capacity to control immediately gratifying temptations to achieve long-term objectives [43]).

### Informing for better autonomy and treatment decision-making

Between the Onset and Tertiary Care (Orthopedic) stages, the patients’ information-seeking behavior demonstrated a strong need for treatment information, which has a cascading effect on their sense-making process; emotional and behavioral responses like pain catastrophizing, pain-related fear learning, and disability [44–47]; sense of control and certainty; decision-making [48–50]; and treatment journey’s length and complexity. This behavior could be an adaptive response to the anxiety and uncertainty [51] arising from symptoms that gradually affected their mobility, physical functions, and daily lives. This is because information facilitates self-efficacy (i.e. perceived control) in self-treatment, which improves pain alleviation, disability, and psychological well-being (e.g. lowering stress, depression and anxiety) [52]. Hence, this study suggests improving community literacy on KOA and related treatment information to reduce uncertainties [53], encourage the seeking of timely treatment; and facilitate decision-making and lifestyle changes [54] for better self-management. Improved community literacy (a term coined by this study as a community’s ability to obtain and understand knowledge on KOA and its treatment information) could help patients to embark on shorter and more satisfactory treatment journeys. Moreover, this could prevent unhelpful beliefs from taking root and avoid a vicious cycle of perceived helplessness, unwillingness to exercise [55], deterioration, and exacerbated pain and symptoms [56, 57]. More importantly, by being informed before doctors’ visits, patients can participate in decision-making during the consultation process to enhance their sense of control [58].

### Communicating for better primary and tertiary care experiences, outcomes, and satisfaction

The patients’ accounts of their experiences and interactions with their primary care physicians are consistent with studies suggesting a simultaneous hierarchical relationship between doctors (i.e. higher hierarchical level) and patients (i.e. lower level) [59–61] and unidirectional communication from physicians to patients [62]. Consistent with Brown [63], patients in this study appear deferential towards and dependent on their Primary Care providers, who are clearly “in a position of professional power” (p.473) by virtue of their position as gatekeepers to treatment and access to subsidized tertiary care. However, the Primary Care providers in this study may not be recognized as experts [63] by their patients when they fail to meet patients’ need for information, advice, and treatment, which could negatively affect their patients’ confidence and trust in them. This means silence is not an option for healthcare professionals. While several reasons could be responsible for Primary Care providers’ lack of communication, perhaps uncertainty [64] is one. It is recommended that Primary Care providers utilize patient-centered communication strategies, which were found to result in significantly positive patient experience [64], for communicating through uncertainty.

Contrary to other studies [65–67], the results of this study found patients seem to attach equal importance to receiving information and care from their doctors. The results showed sufficient communication, especially feedback and education, from healthcare providers to patients has the potential to improve treatment experience and engagement and patients’ confidence in recovery. Conversely, inadequate communication can lead to patients feeling anxious about their condition and recovery, as well as doubt the benefits and their commitment to treatment and its completion. This is because “the patient… is human, fearful and hopeful, seeking relief, help and reassurance” and “tact, sympathy, and understanding are expected of the physician” [68]. And communication is one way for physicians to provide the needed hope, confidence, reassurance, help, and psycho-emotional relief needed by older patients in pain.

The study emphasizes that good healthcare professional-patient rapport needs to be built and maintained at the start of the medical consult for patients to develop confidence and self-efficacy [69], and commitment to treatment. Considering the patients’ needs and limitations faced by healthcare providers, this study suggests healthcare providers could adopt a hybrid agenda-setting-informative communication [70] style. Agenda-setting communication allows one to prioritize selected topics and tasks, direct conversations, and inform and educate patients during time-constrained consultations. Informative communication promotes patient engagement in treatment, positive behavior change, positive beliefs [71], as well as reducing the impact of pain, anxiety, fear avoidance, and kinesiophobia [72]. This would enable healthcare providers to satisfy patients’ expectations and needs and improve treatment experience, while taking advantage of the traditional settings of consultations whereby patients passively trust and follow orders [73].

### Overcoming lack of time and inertia with meaningful, manageable, and adaptable (MMA) care

Participants’ (including those who were satisfied with and had completed their treatment) reported lack of time, and inability to accommodate exercises in daily life [74], which are partially consistent with literature [75] and the larger qualitative study [20] that this study was embedded in, require particular attention. Participants perceive themselves to be too busy [76] and lacked the interest and motivation [77] to engage in home-based exercise after their discharge from physiotherapy especially left without supervision or accountability [78]. Consistent with literature, our findings showed older female patients felt they have little time for exercise as they tend to prioritize the management of their household and caring for their family members [79]. Similarly, those in employment tend to prioritize their work. The perceived “busyness” of older female patients in this study could be compounded by the need to use more time to perform their daily tasks, which become increasingly difficult with advancing age [80]. A lack of household assistance [81] could be another contributing factor.

Pressures and priorities of daily life are known to influence a patient’s motivation and perceived ability to engage in an exercise regime [82–84]. Small changes in one’s condition, needs, support, routine, or even preferences may result in cascading changes to other life domains and workload [85]. This means there is a need for care that is relevant and manageable, yet adaptable to fluctuations of one’s everyday life, motivation, and abilities. Hence, to help patients normalize and integrate exercise into their routine, healthcare providers would need to (1) understand their patient’s social context; (2) help patients realize the need for exercise to be routinized; (3) recognize how life circumstances and priorities can interfere with engagement; (4) and work together on strategies that can make treatment more feasible and adaptable in their busy lives [86]. This process could enable both healthcare providers and patients to achieve mutual understanding in treatment importance and engagement [87]. When exercise treatment becomes a routine, it cultivates both a habit and self-control that helps one to stay engaged and able to delay the immediate gratification of inertia.

### Strength and limitations

This is the first qualitative study that explored and depicted the treatment journey and experience of older KOA patients in a multiracial Southeast Asian context. The study’s qualitative design provided insights into older KOA patients’ knowledge, behavior, thoughts, experiences, and journey, which allowed the study to fulfill its objectives. This study suggests a necessary push for stronger patient engagement, communication and education to improve patient care, and treatment acceptance and engagement. Besides identifying core issues of various touchpoints and levels, recommendations have been made for feasible communication strategies that can be applied during time-constrained consultations while fulfilling patients’ needs and expectations to improve outcomes and treatment engagement and satisfaction. In addition, strategies for patients to cultivate self-control to overcome barriers to effective KOA self-management were suggested. The authors cautioned against generalizing self-control to be strongly associated with Asian individuals as studies have suggested collectivistic (i.e. Asian) societies exhibited stronger behavioral self-control than their individualistic (i.e. Western) counterparts [88]. In this study, inertia and lack of time are individual, rather than collective or cultural, barriers to exercise therapy. Moreover, socioeconomic growth [89] and/or strong confluence of cultures - in the case of Singapore being a society where “East meets West” – could change a society’s individualistic-collectivistic traits.

This study has some limitations. First, the recruitment of participants from the hospital is a sampling limitation. There would be patients who may not go to the hospitals due to mild, sporadic symptoms or personal and/or structural barriers, suggesting that they could be lingering at the Self-treatment or Primary Care stages, or alternate between the two stages. It is likely that these patients could embark on a linear or complex treatment journey should they gain access to the hospital or their condition deteriorate. Second, majority of participants were from a single major ethnicity. This is because the RCT, which the larger a priori qualitative study sampled its participants from, has very few patients from other races. As such, the patients’ experiences may be unique to a particular tertiary care. Many of the themes identified are aligned with current literature. Third, the data presented and discussed in this study was collected in 2020. It is possible that since then patient decisions and experiences and clinical practices, which might have been influenced by COVID-19, could have changed.

## Conclusions

Despite having relatively linear treatment journeys, older KOA patients wanted better and more effective primary care. They identified inadequate communication from primary care doctors and tertiary care physiotherapists as pain points, which could be attributed to systemic issues like short consultation time and high patient loads. Long history of KOA, severe pain, compromised mobility and physical functions, and a lack of awareness of KOA and its treatment options can influence a treatment journey’s length and complexity. Future studies could investigate how older KOA patients are psychosocially affected by longer and more complex treatment journeys.

## Supplementary Information

Below is the link to the electronic supplementary material.

Supplementary Material 1

Supplementary Material 2

## Data Availability

Data is provided within the manuscript as quotes and in Supplementary Tables 3a-c (Additional file 1). The de-identified datasets used and/or analysed during the current study are available from the corresponding author on reasonable request.
