# Supplementary material for: Understanding the treatment journey and experiences of knee osteoarthritis patients receiving standard care in Singapore: a qualitative study
Source: BMC Health Serv Res. 2026 Mar 12;26:557. doi: 10.1186/s12913-026-14333-4 (PMC13097805; doi:10.1186/s12913-026-14333-4)
Supplement: Supplementary file 1 — Supplementary Material 1 [file 12913_2026_14333_MOESM1_ESM.docx]

**Additional file 1**

| **Stages (themes) of a patient journey** | **Framework** | **Sub-themes**  (n = number of unique participants’ mentions) |
| --- | --- | --- |
| **Onset: symptomatic experiences of KOA and its impact on physical function and daily life** | Onset [1–4] and awareness [5, 6]  Symptom deterioration, decreased physical function [3], influence on daily functioning [2, 4], chronic pain is challenging + Associated problems [7] | **Awareness** (n=12) **and perceived severity** (n=9) **of chronic and/or acute symptoms** (n=12)  *I noticed the pain in my knee when I started walking, even worse when I had to climb up or down stairs to the extent that half the time, I'm afraid of falling downstairs... sometimes when I'm walking on flat ground, there is pain.* – P11  *I was trying to climb the steps in my house and then suddenly it hurt and I just couldn’t move!* – P14  *I thought arthritis shouldn’t be so severe as to I can’t even walk for half the day... my arthritis is so severe, suddenly just attack... I thought that I won’t be able to walk anymore.* – P22  **Influence on physical and daily function** (n=19) (including impact on mobility (n=16))  *When holding grocery with two hands, I got problem going down stairs. From the carpark to my lift lobby, there's quite a long flight of stairs… I walk slowly, slowly down the stairs, because if I fall down with the bag of groceries, then I'll be in the hospital again for a different reason.* – P11  *Before the knee pain I walk a lot. I can* (brisk) *walk for 4-5 hours.* – P13 |
| **Sense-making and seeking knowledge on cures** | Rationalizes [4]  Becoming your own expert can be hard work [8, 9], Lack of knowledge [2] | **Caused by wear and tear, falls, or injury** (n=13)  *I think it’s just with age and all that… when you age, all these are issues that you have to overcome or accept.* – P14  *Quite a serious one... these two knees landed on the floor. My knees swelled up like a tennis ball. I think it's because of the fall… if you don’t have accident, nothing will just come like that.* – P07  **Lack of knowledge** (n=7)  *I don’t know. I really cannot find any solution. Looks like only way is to cut. But I try to get information.* – P21  **Mixed stories about surgery** (n=16)  *My neighbor the Indian guy went for knee operation two years ago. Now he can't walk already, must use a walker. That’s why if you can take the pain, don’t go for operation.* – P07  *I talk to friends that had operation. To them they said nothing what, very easy, they operate! I’m thinking in my mind, sure. And I talked to another older one he said he’s about 80 already, after the operation he felt good.* – P04 |
| **Self-treatment: self-efficacy in using and evaluating a variety of strategies** | Simple problem (initial presentation, movement therapies, underlying cause, no easy fix) [1],  Self-management [1] | **Self-managed with topical applications (n=15), supplements (n = 15), and exercise and physical activity** (n=12)  *I sit down and then use some ointment to rub on my knee, hoping that I will be able to stand up and walk.* – P22  *I have been eating glucosamine for about three to four years. The private doctor told me that it's useless and that I can throw it away, I don’t have to eat it.* – P10  *I start brisk walking, I do swimming... I thought that my health is my own responsibility… it’s not so painful anymore* – P22  **Avoiding painkillers due to perceived harm** (n=10) **but medicate when necessary** (n=9)  *People say you take a lot, your kidney will spoil. That’s why* (I) *let it pain* – P02  *Morning before I go to work, I will take the Panadol. Then evening, I cannot tahan* (tolerate) *already, I will take. If I can take it, I never take at all.* – P16  **TCM is ineffective, unreliable, and not a cure** (n=17)  *TCM cannot really do much where bones are concerned… I don’t think it can repair the wear and tear.* – P09  *It’s a hassle... The effect would last about two to three days after acupuncture... I don’t want a temporary cure. I just want something that really can solve my problem*. – P22  *I tried for four weeks; one week once. There is no effect. Consult and receive medicine only. Nothing much, he also doesn’t tell you much… it won’t get better, consult for what? It’s just spending of money!* – P15 |
| **Primary care: seeking “help” but felt dismissed** | Seeks help in primary care [4], screening [5, 6], and the emerging need for an orthopedic evaluation [3] | Seeks help at primary care (n=19): X-ray done (n=6); received analgesia (n=8); received glucosamine and/or supplements (n=5); prescribed or advised to exercise (n=3); received diagnosis (n=5); did not receive screening, diagnosis, information, and treatment (n=8)  Seeks help at a hospital’s Emergency Department (n=4)  Seeks help at private hospital (n=4)  Extended treatment journey [having ≥ 4 touchpoints] (n=6), repeated polyclinic visits to receive referral (n=3) |
| **Tertiary care (orthopedic consultation): decided surgery is a last resort** | Orthopedic diagnosis [3, 5, 6], and shared surgical decision-making [1–3, 8] | X-ray done (n=11); received education and/or advice (n=10); received diagnosis (n=7)  **No desire for surgery or intends to avoid surgery** (n=19)  *Better don’t go for any operation… I told my wife I don’t want to touch anything, just leave the pain like that.* – P07  *I feel that when you reach a certain age, you heal slower. It’s not advisable to cut if you can avoid it. I am not for surgery, and I don’t want to go through that pain.* – P14 |
| **Tertiary care (physiotherapy): positive experiences, symptomatic improvement, and treatment adherence** | Treatment [2, 4–6] | **Treatment adherence** (n=22)  *After the first session, talking to the physiotherapist, I suppose the amount that I can recover will most probably be equivalent to the amount of effort I put into the exercises even when I'm not here. That means at home I need to do these exercises.* – P11  **Positive experiences with** (n=19) **and gaining confidence from therapist** (n=10)  *They are very nice. She* (physiotherapist) *knows that I'm in pain, "okay take your time, take your time". So, I’ll take my time. She’ll never force you to do something you cannot do, which I enjoy! Because of that, I try my very best to make myself better. It is motivating and encouraging because the physiotherapy helps to boost your energy. Like you do this "ah! I can really go better, I can walk faster, I feel lighter", gives you the oomph!* – P16  *The physiotherapist gives the confidence, and do it in a practical way that is really proven, and there is someone to guide me. If you ask me to do on my own, I don’t think so. Someone guiding me then I have the confidence to heal.* – P03  **Symptomatic improvement** (n=13) **from exercises that are simple, personalized, and instrumental to recovery** (n=15)  *I can see that got improvement because I don't need to use the walking stick and I can walk the stairs faster... more stable without holding onto the railing.* – P24  *I was very happy because through physiotherapy, I don’t have to take any medicine… I feel that it's good. Actually, physiotherapy is also very effective, you just need a few moves and it can be very effective.* – P12 |
| **Tertiary care (treatment discontinuation): dissatisfaction with brief sessions, lack of communication, and prescribed exercises** |  | **Treatment not worth continuing** (n=11)  *After the second session, I don't want to come anymore because I feel I can do those exercises on my own*. – P02  *We come all the way here, the therapist only teach us 1 or 2 exercise, and settle within 20 min or half an hour. I think it's a waste of time for us travelling so far here.* – P24  **Lack of communication from therapist** (n=6)  *After I’m done with the exercises, that’s it, I go home already. No connectivity, no further feedback advice or concern.* – P22  **Exercises are either basic and repetitive, or difficult and painful** (n=14)  *What they taught me are basic. If I had to come every week for 10 weeks, if I have to do the same thing, I will get bored.* – P11  *The first session is very mild... ask me to climb up the staircase, but when I climb up the staircase, go down, there's a problem... Once I even fall down because it's so painful.* – P19 |
| **Post-treatment**  **Improvement to physical functioning and pain**  **Adhering and self-managing with exercise and physical activity**  **Motivations behind regular exercise and physical activity**  **Active acceptance and positive mindset**  **Barriers: lack of time and inertia** | Self-manage without health professional involvement [4]  Control or Remission [5, 6]  Adherence [5, 6]  Learning, accepting, and living with the illness [9] | **Experienced improvement** **to pain** (n=17)**;** **physical functions** (n = 12)**;** **and overall health** (n=8)  … I put in effort… once cured, when I play *(badminton)*, I really dash for the ball… – P05  **Adherence and self-discipline** (n=17)  *Continue doing more, don’t be lazy. Laziness will only hurt yourself. Doctor tells you to go and do, you must go and do, do until you improve (and you) will happy already.* – P17  *I will do every day, cannot be lazy. One day never do, I feel I got something wrong with me, so I will do, it's a habit.* – P02  **Self-managing with exercise or physical activity** (n=15)  *Now I already know what to do when it hurts, I know those few exercises. I will use those actions to help myself. I find those exercises to be helpful.* – P18  **(Self-)motivated to improve and avoid pain** (n=16)  *You must persist in exercising frequently, if you stop, it will return. To avoid hurting again, I tell myself I must continue exercising.* – P18  *… I always have this thinking that since people make effort to help me, I cannot don't do anything… that's why I try my best to do more.* – P24  **Change in mindset** (n=8)  *No choice. I think don’t think too much… Now my life is okay, I feel very happy, it's okay that the knee creaks... don’t be so greedy, right? Pain is natural, everybody got problem, only less or more… That’s why compared to other people, my life is better than theirs... How much the Lord gives you, take it*. – P02  **Busy or perceived lack of time** (n=1**3**)  *When I recover, I* (went) *back to my normal life, fully focused on my work, no time for exercise.* – P03  *Because of my shift duty, I can’t do every day. Sometimes, the whole day, I will be at work.* – P06  **Laziness** (n=6)  *Sometimes very lazy… you don't feel like doing anything, then doing less and less… it's very dull, you don't feel like doing anything.* – P19 |

Supplementary Table 3a. Overview of subthemes, categories, and themes

| **Hoping to recover via physiotherapy to avoid surgery** | “You all taught me to exercise… I think no need to operate, slowly the condition will not be that bad. Because if you operate it is very troublesome” (P17)  “Best not to undergo surgery because there will be side effects. I thought of starting with physiotherapy. Try and if it fails then of course consider surgery” (P18)  “When I came to see the specialist, I know very sure can avoid the surgery. So, I can just cure myself with the exercises... If go for surgery, I don’t think so our walking will be the same… better to avoid surgery” (P06)  “I hope no need to do surgery, of course I hope can go for treatment, the physiotherapist help me try first, if cannot then will say again” (P20)  “I feel I can help myself… hopefully it (physiotherapy) can help me to avoid that (surgery)” (P09) |
| --- | --- |
| **Avoiding surgery due to lack of guaranteed positive outcomes** | “Surgery also no guarantee to be okay. After the surgery may not help and surgery is not cheap. So, I though it's not very very serious, just let it go” (P08)  “I know all surgery carry risk. So it doesn’t guarantee that it will be a success right? So I never considered that at all” (P22)  “Knee surgery there’s no guarantee that you will have a perfect operation because I do have friend that doesn’t work for him” (P04)  “I don’t want to risk having to go through that pain and then in the end of the day it may or may not help” (P14)  “Surgery's a long process, after that you take a couple of months (to recover) and then maybe no good and have to suffer all the pain. My age ah, better not to go through all these” (P19) |
| **Considered or urged to undergo surgery** | “I already give up, I want to go for operation because I really cannot take it anymore… the pain, when it comes, I really got no confidence really because I really cannot tahan (tolerate)… my daughter and son told me, "mummy, you go for operation"… My son want me to operate. Because the doctor talk to him, he said "I think you need to operate, okay?” (P16)  “Most of my friends always tell me "no point going for physiotherapy, just go straight for a kneecap replacement" (P03)  “it was so painful that I thought of going for surgery to get rid of the suffering” (P20) |

Supplementary Table 3b. Overview of subthemes, categories, and themes pertaining to surgery considerations

| **Preferring other exercises over physiotherapy** | “I still go for my run. I go gym, I do a lot of squats… previously I don’t do weightlifting squat, but after that I do weightlifting squat. I don’t want the pain to come back again, so I'm training my muscles” (P03)  “Physio(therapist) tells me you have to do it (physiotherapy) every day. So tried to do every day, and then it helps… After that I try to do my morning walk. Eh? I can walk more, it don’t hurt so much!... After walking more I stopped doing physio. I think physio helps but walking helps more… after my walk I feel so much better because you sweat a lot then you find that you are more healthy, more awake, more energetic! Because walking will strengthen myself… so I continue with my walks” (P04)  “At home I do light exercise… like dumbbell, stretches, and also a bit of knee exercise for about ten minutes… I think Zumba and chair exercise also help me to improve my leg… I play badminton every week. Every Thursday I play badminton” (P05)  “I did work out because my park there they have this treadmill… I got this watch, so after dinner I will just go round my park for 10,000 steps... Usually Monday to Friday I am more active. I will walk around my park in the morning, and then in the evening after dinner” (P23) |
| --- | --- |
| **Keeping physically active** | “My daily work includes cleaning, moping the floor... cleaning the toilets and stairs… all these are a form of movement… More or less, these things accumulated, but it's not the vigorous type” (P07)  “I know it's an ongoing thing... strengthening will not be done on it's own without me doing anything... I will sit up and down. When I'm sitting, I will lift my knee up, stretch, or do some exercises. Not to be totally sedentary” (P11)  “I take regular morning exercise walk through the park connector for about half an hour. That is useful, doesn’t affect me at all” (P14)  “I’ll do exercise at home, lie down then I lift up my leg and do pedaling. When I work, I sit down, listen to music, then I will move my leg up, I follow the beat of the song” (P16)  “I do (exercise) myself at home. One day twice, one in the morning and then in the evening. One week at least 3 times, morning and night” (P08) |
| **Self-managing pain with exercise and physical activity** | “When my leg start to hurt, I know it's because I did lesser exercises, so I’ll quickly stretch and move about… I feel that as long as I follow the physiotherapy exercises, where hurts, I can stretch the area, it is a cure without medicine. So when I'm at home, I can stretch the areas that are painful” (P12)  “I know what to do when it hurts, I know and will use those few exercises to help myself. Those exercises are helpful” (P18)  “Sure pain, then later, slowly I do exercise, I don’t feel so much pain” (P02)  “If I find that my leg is stiff, so better do something to regain myself” (P16) |

Supplementary Table 3c. Overview of themes pertaining to self-managing post-treatment


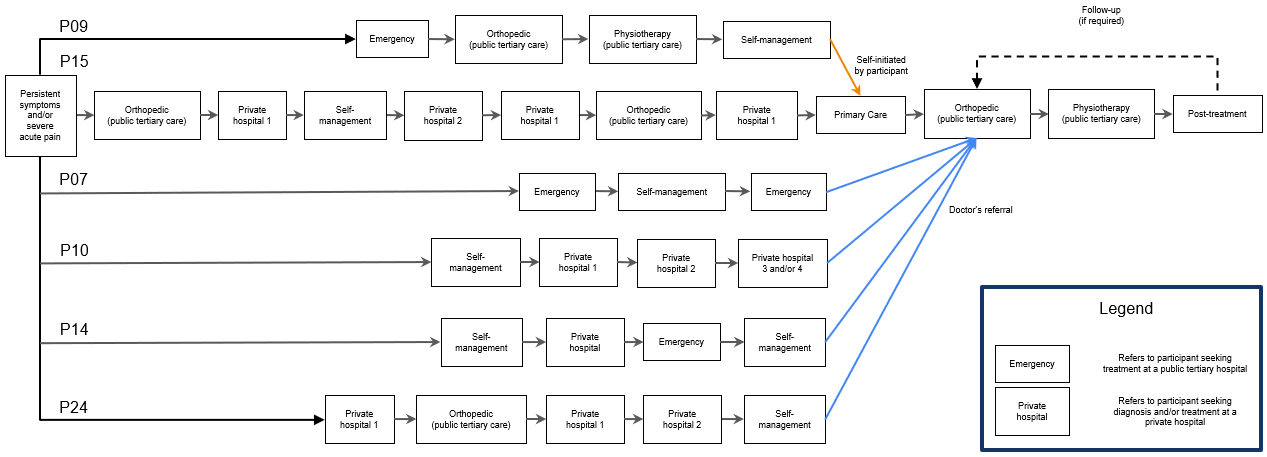
 Supplementary Figure 1. Mapping complex treatment journeys

**References**

1. Morlion B, Finco G, Aldington D, Überall M, Karra R. Severe chronic low back pain: patient journey from onset of symptoms to strong opioid treatments in Europe. Pain Manag. 2021;11:595–602.

2. Van Schalkwijk DL, Widdershoven JWMG, Elias-Smale S, Hartzema-Meijer M, Den Oudsten BL, Saltman J, et al. ShareHeart: A patient journey map of patients with ischemia and non-obstructive coronary artery disease based on qualitative research. J Clin Nurs. 2023;32:3434–44.

3. Brembo EA, Kapstad H, Eide T, Månsson L, Van Dulmen S, Eide H. Patient information and emotional needs across the hip osteoarthritis continuum: a qualitative study. BMC Health Serv Res. 2016;16:88.

4. Durham J, Steele J, Moufti MA, Wassell R, Robinson P, Exley C. Temporomandibular disorder patients’ journey through care. Community Dent Oral Epidemiol. 2011;39:532–41.

5. Bharatan T, Devi R, Huang P-H, Javed A, Jeffers B, Lansberg P, et al. A Methodology for Mapping the Patient Journey for Noncommunicable Diseases in Low- and Middle-Income Countries. J Healthc Leadersh. 2021;Volume 13:35–46.

6. Bahlas SM, Chami ZE, A Amir A, Khader S, Bakir M, Arifeen S. A Semi-systematic Review of Patient Journey for Chronic Pain in Saudi Arabia to Improve Patient Care. Saudi J Med. 2021;6:47–56.

7. Blackbeard D, Aldous C. Interviewing older men at an interdisciplinary pain clinic: the journey to chronic pain and treatment experience. South Afr J Psychol. 2023;53:576–88.

8. Barker KL, Toye F, Seers K. A synthesis of qualitative research to understand the complexity behind treatment decision-making for osteoarthritis. Osteoarthr Cartil Open. 2023;5:100355.

9. Kane S, Joshi M, Desai S, Mahal A, McPake B. People’s care seeking journey for a chronic illness in rural India: Implications for policy and practice. Soc Sci Med. 2022;312:115390.
