# Supplementary material for: Understanding the treatment journey and experiences of knee osteoarthritis patients receiving standard care in Singapore: a qualitative study
Source: BMC Health Serv Res. 2026 Mar 12;26:557. doi: 10.1186/s12913-026-14333-4 (PMC13097805; doi:10.1186/s12913-026-14333-4)
Supplement: Supplementary file 2 — Supplementary Material 2 [file 12913_2026_14333_MOESM2_ESM.docx]

| **Item number** | **Guide questions/description** | **Reported on page number** |
| --- | --- | --- |
| **Domain 1: research team and reflexivity** | | |
| **Personal characteristics** | | |
| 1. Interviewer/facilitator | Which author/s conducted the interview or focus group? | 4 |
| 2. Credentials | What were the researcher’s credentials (e.g. PhD, MD)? | 4 |
| 3. Occupation | What was their occupation at the time of the study? | 4 |
| 4. Gender | Was the researcher male or female? | 4 |
| 5. Experience and training | What experience or training did the researcher have? | 4 |
| **Relationship with participants** | | |
| 6. Relationship established | Was a relationship established prior to study commencement? | 4 |
| 7. Participant knowledge of the interviewer | What did the participants know about the researcher (e.g. personal goals, reasons for doing the research)? | 4 |
| 8. Interviewer characteristics | What characteristics were reported about the interviewer/facilitator (e.g. bias, assumptions, reasons and interests in the research topic)? | 4 |
| **Domain 2: study design** | | |
| **Theoretical framework** | | |
| 9. Methodological orientation and Theory | What methodological orientation was stated to underpin the study (e.g. grounded theory, discourse analysis, ethnography, phenomenology, content analysis)? | 4 |
| **Participant selection** | | |
| 10. Sampling | How were participants selected (e.g. purposive, convenience, consecutive, snowball)? | 4 |
| 11. Method of approach | How were participants approached (e.g. face to face, telephone, mail, e-mail)? | The process was described in another embedded study |
| 12. Sample size | How many participants were in the study? | 4 |
| 13. Non-participation | How many people refused to participate or dropped out? Reasons? | 4 |
| **Setting** | | |
| 14. Setting of data collection | Where was the data collected (e.g. home, clinic, workplace)? | 4 |
| 15. Presence of non-participants | Was anyone else present besides the participants and researchers? | 4 |
| 16. Description of sample | What are the important characteristics of the sample (e.g. demographic data, date)? | 7 (Table 2) |
| **Data collection** | | |
| 17. Interview guide | Were questions, prompts, guides provided by the authors? Was it pilot tested? | Yes. Page 4 |
| 18. Repeat interviews | Were repeat interviews carried out? If yes, how many? | n/a, not relevant to this method as participants were contacted only once for the study |
| 19. Audio-/visual-recording | Did the research use audio or visual recording to collect the data? | 4  Interviews were audio-recorded. Data collection was described in another embedded study |
| 20. Field notes | Were field notes made during and/or after the interview or focus group? | 4 |
| 21. Duration | What was the duration of the interviews or focus group? | 4 |
| 22. Data saturation | Was data saturation discussed? | 5-6 |
| 23. Transcripts returned | Were transcripts returned to participants for comment and/or correction? | 4 |
| **Domain 3: analysis and findings** | | |
| **Data analysis** | | |
| 24. Number of data coders | How many data coders coded the data? | 5 |
| 25. Description of the coding tree | Did authors provide a description of the coding tree? | Additional File 1 (Supplementary Tables 3a, 3b, and 3c) |
| 26. Derivation of themes | Were themes identified in advance or derived from the data? | 5 |
| 27. Software | What software, if applicable, was used to manage the data? | 5 |
| 28. Participant checking | Did participants provide feedback on the findings? | n/a, not relevant to this method as participants were contacted only once for the study |
| **Reporting** | | |
| 29. Quotations presented | Were participant quotations presented to illustrate the themes/findings? Was each quotation identified (e.g. participant number)? | 9-13 |
| 30. Data and findings consistent | Was there consistency between the data presented and the findings? | 9-13 |
| 31. Clarity of major themes | Were major themes clearly presented in the findings? | 9-13 |
| 32. Clarity of minor themes | Is there a description of diverse cases or discussion of minor themes? | 9-13 |
